# *Strongyloides* Hyperinfection Syndrome and Disseminated Disease with Negative Serology

**DOI:** 10.4269/ajtmh.24-0460

**Published:** 2024-10-01

**Authors:** Ashton D. Hall, Divya Salibindla, Keith M. Luckett

**Affiliations:** ^1^ Division of Infectious Diseases, Department of Internal Medicine, University of Cincinnati College of Medicine, Cincinnati, Ohio;; ^2^ Department of Pathology and Laboratory Medicine, University of Cincinnati College of Medicine, Cincinnati, Ohio

**Figure 1.**
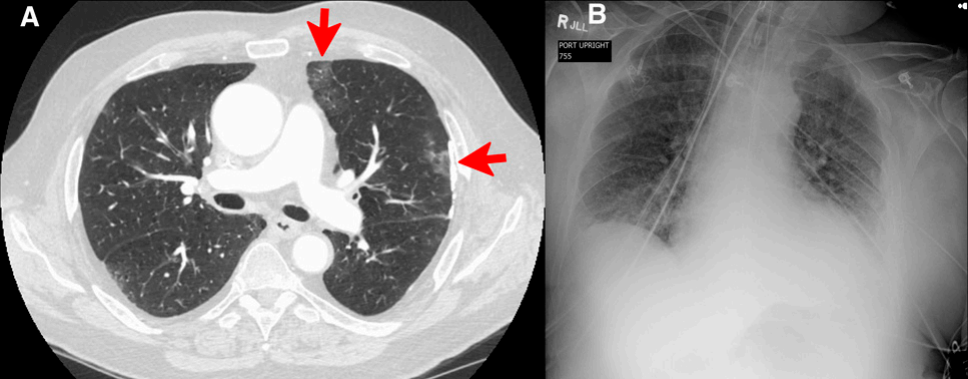
Computed tomography pulmonary angiography at the patient’s initial presentation revealed patchy ground-glass opacities in the upper lobes (red arrows, **A**). Chest X-ray 2 days before death revealed bibasilar airspace opacities, especially prominent within the left lung base and obscuring the left heart border, with decreased left lower lobe aeration (**B**). Courtesy of M. B. Burch at the University of Cincinnati.

A 65-year-old man with a history of stage IV follicular lymphoma, who discontinued rituximab monotherapy 3 weeks ago, presented with acute hypoxia and dyspnea. Laboratories revealed leukocytosis (12.6 × 10^3^ cells/*µ*L) with chronic eosinophilia (49%; 6,174 cells/*µ*L). Initial concern was for eosinophilic pneumonitis or a chronic obstructive pulmonary disease exacerbation. Blood cultures and a respiratory viral panel were negative. Computed tomography pulmonary angiography revealed ground-glass opacities in the upper lobes (Figure 1). He was discharged after 5 days with levofloxacin and dexamethasone.

**Figure 2.**
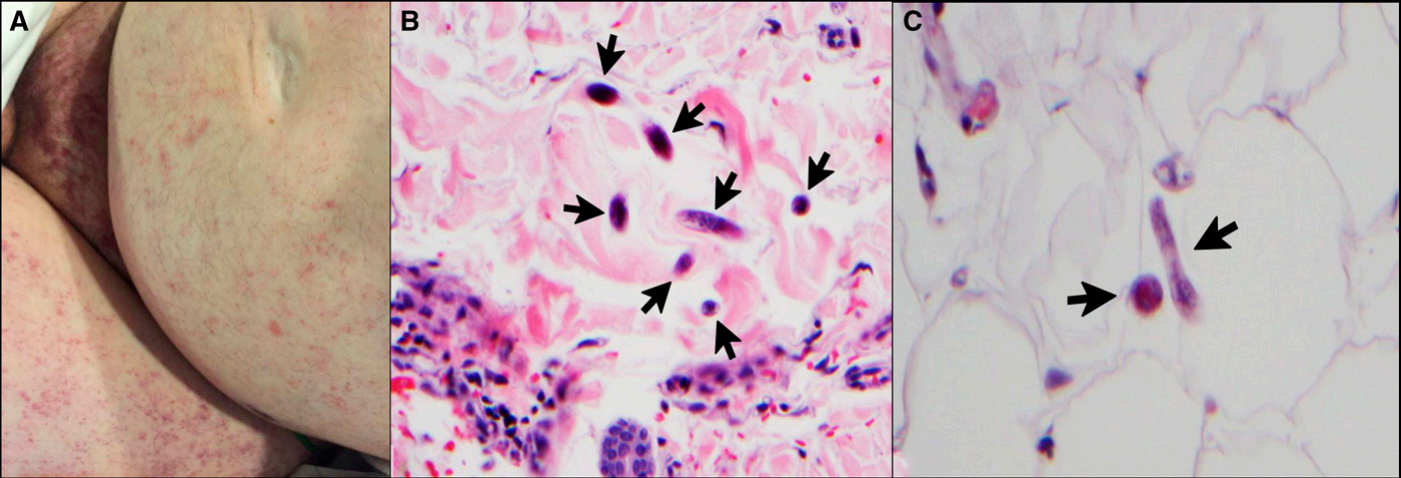
Skin examination revealed numerous 1–2-mm round, nonblanching purpuric macules involving the groin and thighs with a few well-defined, slightly scaly, red-purple papules and plaques (**A**). Hematoxylin and eosin stain of skin biopsy specimens from the left hip and left abdomen at 40× magnification showed filariform larvae in coronal and transverse sections (black arrows, **B** and **C**). Courtesy of K. E. Spicknall at the University of Cincinnati.

**Figure 3.**
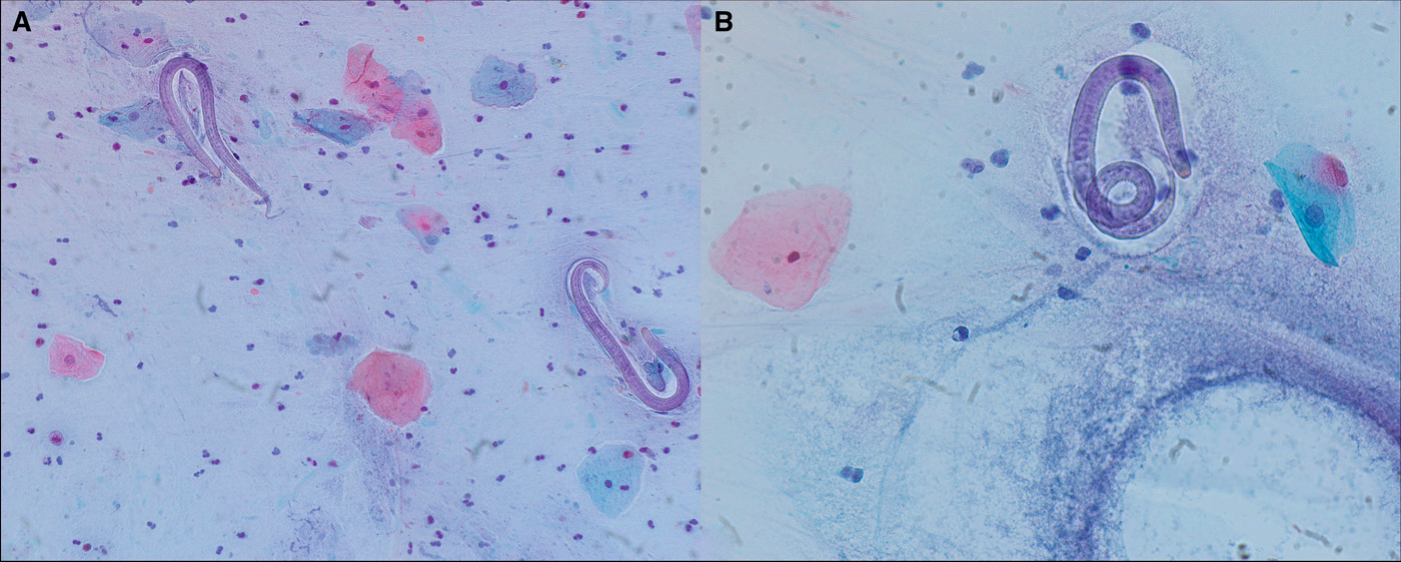
Sputum culture with Papanicolaou staining on hospital day 7 of his second admission revealed many L3 (filariform) larvae consistent with *Strongyloides stercoralis* at 20× and 40× magnification (**A** and **B**). Courtesy of D. Salibindla at the University of Cincinnati.

He presented 2 weeks later with dyspnea, diarrhea, abdominal pain, and a pruritic rash on the groin and legs. He denied a history of serpiginous skin eruptions but reported fishing in a reservoir 6 weeks ago. Laboratories revealed leukocytosis (21.6 × 10^3^ cells/*µ*L) without eosinophilia. *Toxoplasma* serology and blood cultures were negative. Levofloxacin, prednisone, albuterol, and tiotropium were started without clinical improvement. The patient’s code status was changed to do-not-resuscitate and do-not-intubate, which precluded bronchoscopy. Skin biopsy and sputum culture demonstrated many filariform larvae morphologically consistent with *Strongyloides stercoralis* (Figures 2 and 3). Stool ova and parasite studies were not done. Steroids were tapered, and he was started on ivermectin (215 *µ*g/kg/day). He withdrew from life-saving measures owing to respiratory and renal failure. *Strongyloides* IgG serology was negative on samples collected 3 weeks before his first hospitalization and 1 day before death. The patient had several epidemiologic risk factors for *Strongyloides* hyperinfection syndrome (SHS), including a 10-year history of non-Hodgkin lymphoma, multiple courses of steroids, and recent rituximab therapy.

*Strongyloides stercoralis*, or threadworm, is a soil-transmitted helminth endemic to tropical, subtropical, and warm temperate climates, including Appalachia and rural parts of the southeastern United States.^1^ Approximately 30–100 million people worldwide have strongyloidiasis, although an accurate estimate of disease prevalence is skewed by asymptomatic infections.^1^ *Strongyloides* hyperinfection syndrome often occurs in immunocompromised patients through autoinfection, where filariform larvae penetrate the intestinal mucosa or perianal skin, increasing host worm burden, morbidity, and mortality.^2^ At-risk populations for SHS include transplant recipients, patients with hematologic malignancies, and patients receiving immunosuppressive medications.^2^ *Strongyloides* serology is sensitive (88–98%) but may be unreliable early in the course of illness among vulnerable populations.^3^^,^^4^ Patients at increased risk of SHS may benefit from empiric treatment, even when serologic testing is negative, because of its high mortality rate (15–87%) and limited physician experience with the disease outside endemic areas.^2^^,^^5^ Duration of ivermectin (200 *µ*g/kg/day) relies on negative stool or sputum studies for 14 days.^6^
